# Comparative study of the inhibitory effect on bone erosion progression with denosumab treatment and conventional treatment in rheumatoid arthritis patients: study protocol for an open-label randomized controlled trial by HR-pQCT

**DOI:** 10.1186/s13063-019-3589-8

**Published:** 2019-08-13

**Authors:** Naoki Iwamoto, Shuntaro Sato, Remi Sumiyoshi, Ko Chiba, Nanami Miyamoto, Kumiko Arinaga, Makiko Kobayashi, Hiroshi Yamamoto, Makoto Osaki, Atsushi Kawakami

**Affiliations:** 1 Department of Immunology and Rheumatology, Division of Advanced Preventive Medical Sciences, Nagasaki University Graduate School of Biomedical Sciences, 1-7-1 Sakamoto, Nagasaki, 852-8501 Japan; 2 Nagasaki University Hospital, Clinical Research Center, 1-7-1 Sakamoto, Nagasaki, 852-8501 Japan; 3 Department of Orthopedic Surgery, Nagasaki University Graduate School of Biomedical Sciences, 1-7-1 Sakamoto, Nagasaki, 852-8501 Japan; 4 Medical Science Department, Daiichi Sankyo Co., Ltd, 3-5-1 Nihonbashi honmachi Chuo-ku, Tokyo, 103-8426 Japan

**Keywords:** Rheumatoid arthritis, RANKL, Denosumab, Bone erosion, Bone micro-architecture, HR-pQCT

## Abstract

**Background:**

Rheumatoid arthritis (RA) is a chronic inflammatory disease of the joints, causes joint destruction, and leads to physical disability. Advances in the treatment of RA, such as biologic disease-modifying anti-rheumatic drugs (DMARDs), have provided better clinical outcomes, including the achievement of remission for patients with RA, but some patients cannot receive these treatments because of their side effects and high cost, and not all patients achieve remission. Although the efficacy of denosumab, which is a human IgG2 monoclonal antibody with a high affinity for the receptor activator of nuclear factor kappa B (RANK) ligand (RANKL), in the treatment of RA has been reported in clinical trials, the efficacy of denosumab in both preventing joint destruction and improving disease activity has not been evaluated in a real-world setting.

**Methods/design:**

This open-label, randomized, parallel-group study will compare the continued use of conventional synthetic DMARDs (csDMARDs) alone with the combined use of csDMARDs and denosumab in patients whose RA is treated with csDMARDs. In total, 44 patients with RA will be randomly assigned to receive additional treatment with denosumab or to continue RA treatment without additional denosumab. The duration of the intervention will be 12 months. To analyze bone erosion and bone micro-architecture precisely, high-resolution peripheral quantitative computed tomography (HR-pQCT) will be performed every 6 months. The primary endpoint is changes in the depth of bone erosion as measured by HR-pQCT from baseline to 6 months. Important secondary endpoints are the changes from baseline in the width and volume of bone erosion as measured by HR-pQCT and changes from baseline in the depth of bone erosion at 12 months. Changes in bone micro-architecture will also be analyzed as an exploratory endpoint.

**Discussion:**

The results of this study are expected to provide strong evidence regarding the usefulness of denosumab for the treatment of RA. Moreover, by using HR-pQCT, this study will also reveal the effect of denosumab not only on bone erosion but also on bone micro-architecture.

**Trial registration:**

This study was registered with the University Hospital Medical Information Network Clinical Trials Registry as UMIN000030575 on December 26, 2017.

**Electronic supplementary material:**

The online version of this article (10.1186/s13063-019-3589-8) contains supplementary material, which is available to authorized users.

## Background

Rheumatoid arthritis (RA) is a chronic autoimmune disease with unexplained immune abnormalities, characterized by joint inflammation resulting in bone destruction, which eventually leads to physical disability and premature death. Biologic disease-modifying anti-rheumatic drugs (bDMARDs) and Janus kinase (JAK) inhibitors are able not only to suppress RA-related joint inflammation but also to prevent structural joint damage. Thus, their introduction has caused a paradigm shift in RA treatments from aiming merely to relieve inflammatory symptoms such as joint swelling and pain to aiming even to achieve structural remission. In practice, however, safety concerns prevent some patients with RA from using bDMARDs and JAK inhibitors. The high costs of bDMARDs and JAK inhibitors may also limit patient access to these drugs for economic reasons. Patients declining bDMARDs and JAK inhibitors for these or other reasons may fail to adequately control their disease activity, and failure to receive an aggressive treatment allows joint destruction to progress.

Denosumab is a human IgG2 monoclonal antibody with high affinity for the receptor activator of nuclear factor kappa B (RANK) ligand (RANKL). RANKL binds to RANK expressed on the surface of resorbing osteoclasts and their progenitor cells, thereby inducing osteoclast differentiation, maturation, and activation and promoting bone resorption. In inflamed joints of patients with RA, activated T and B cells infiltrate and synovial fibroblasts undergo abnormal proliferation. Since these cells express high levels of RANKL [1], they may induce bone destruction around RA joints [2]. Denosumab, through its selective binding to RANKL, inhibits RANK–RANKL interaction, thereby inhibiting the bone resorption by osteoclasts. A phase 2 trial conducted in Japan called DRIVE, which evaluated the efficacy of denosumab in RA, demonstrated the preventive effect of denosumab on the progression of bone erosion [3]. However, until now, little information has been available on the effectiveness of the drug in a real-world setting because clinical trials are conducted in a selected population with strict restrictions on concomitant treatments.

High-resolution peripheral quantitative computed tomography (HR-pQCT) is a new technique with high spatial resolution that enables us to assess the micro-architecture of cancellous and cortical bones that cannot be assessed by conventional x-ray examinations. HR-pQCT can also collect data with high reproducibility at low radiation doses and thus is expected to become a useful tool for elucidating the pathologic basis of a disease and evaluating drug efficacy for the disease. In patients with RA, HR-pQCT can detect bone erosion and joint space narrowing with high sensitivity as compared with conventional x-ray and magnetic resonance imaging (MRI) [4, 5]. Moreover, HR-pQCT can analyze detailed features of bone erosion such as the depth, volume, and width, thus making it possible to quantitatively assess bone erosion. For example, a recent report which evaluated the efficacy of tumor necrosis factor alpha (TNFα) inhibition therapy using HR-pQCT revealed that changes in erosion volume were significantly correlated with changes in the disease activity score in 28 joints (DAS28) [6].

The major aims of this study are to evaluate the efficacy of denosumab in RA for both preventing joint destruction and improving disease activity in a real-world setting and to analyze the effect of denosumab on bone micro-architecture by using HR-pQCT. The results of this study would help the optimal use of denosumab in the treatment of RA.

## Methods/Design

### Study design

**Fig. 1:**
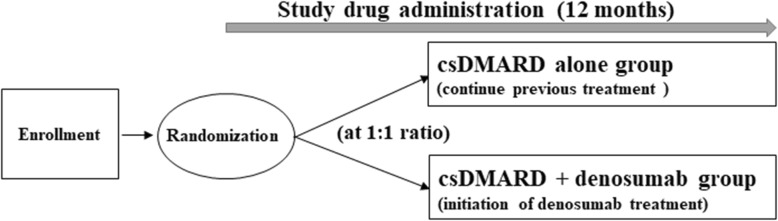
Study design. *Abbreviation*: *csDMARD* conventional synthetic disease-modifying anti-rheumatic drug.

The protocol was designed in accordance with the guideline [7] (Additional file 1). The present study is an open-label, randomized, parallel-group study aiming to evaluate the efficacy and safety of addition of denosumab to any conventional synthetic DMARDs (csDMARDs) (methotrexate, tacrolimus, leflunomide, bucillamine, sulfasalazopyridine, and iguratimod) as compared with continued use of the csDMARDs alone in patients whose RA is treated with a csDMARD. The study will be conducted at a single center (Nagasaki University Hospital). In total, 44 patients with RA will be randomly assigned to receive additional treatment with denosumab or to continue RA treatment without additional denosumab. The duration of the intervention is 12 months. The study design is summarized in Fig. 1.

The study was approved by the institutional review board (IRB) of Nagasaki University (IRB approval number: 18011517). The study is registered in the University Hospital Medical Information Network Clinical Trials Registry (http://www.umin.ac.jp/ctr/) as UMIN000030575. We will conduct the study in accordance with the principles of the Declaration of Helsinki and the Japan Good Clinical Practice guidelines and in compliance with the Ethical Guides for Medical Studies in Human Subjects (promulgated on December 22, 2014), the Act on the Protection of Personal Information and related regulatory notifications, and this clinical study protocol.

### Inclusion criteria

Patients must meet all of the following requirements to be considered for entry into the study: (1) diagnosis of RA according to the American College of Rheumatology (ACR) RA Classification Criteria 1987 Revision or ACR/European League Against Rheumatism (EULAR) 2010 RA Classification Criteria [8, 9], (2) at least moderate or low disease activity, (3) treatment with any csDMARDs, (4) progressive bone erosion confirmed by x-ray, MRI, or musculoskeletal ultrasound imaging, and (5) age of at least 20 years.

### Exclusion criteria

To prioritize the treatment of osteoporosis, we will exclude patients who have not been treated for osteoporosis despite complication of osteoporosis. To avoid influencing the efficacy assessments, we will also exclude patients who are concomitantly receiving the following drugs: intravenous bisphosphonate, parathyroid hormone analogue, denosumab, any bDMARDs, or a JAK inhibitor. The other major exclusion criteria are as follows: (1) the concurrent use of a corticosteroid equivalent to more than 10 mg/day of prednisolone, (2) a history of hypersensitivity to any ingredient of denosumab, (3) hypocalcemia, (4) pregnancy, and (5) being judged by the clinical investigator as an inappropriate patient for the study.

### Randomization

After the acquisition of written informed consent and the completion of screening measurements, eligible patients will be randomly assigned at a 1:1 ratio to receive denosumab in addition to current csDMARDs (csDMARDs with denosumab) or to continue using current csDMARDs alone (csDMARD therapy alone). Randomization will be performed by using the minimization method with stratification by anti-cyclic citrullinated peptide (anti-CCP) antibody (positive versus negative) and sex. Subjects will be enrolled centrally by using an interactive voice/Web response system.

### Intervention

The subjects randomly assigned to the csDMARDs with denosumab group will receive denosumab every 6 months during the study period. As in standard of care, these subjects will receive daily vitamin D/calcium or active vitamin D supplements. In this group, subjects who have received any oral bisphosphonate or any selective estrogen receptor modulator before entry into the study (or both) must discontinue these drugs before receiving the first dose of denosumab. For those who have received any oral bisphosphonate before entry into the study, there must be an interval between the last dose of the bisphosphonate and the first dose of denosumab (given at the month-0 visit) that is longer than the standard dosing interval for the bisphosphonate indicated on its label. For example, the first dose of denosumab should be administered one day after the last dose of the bisphosphonate or later if the bisphosphonate is to be administered once daily and on the 8th day or later if the bisphosphate is to be administered once weekly.

In the subjects randomly assigned to the csDMARD therapy alone group, the current therapy for RA, in principle, will be continued without an addition of denosumab throughout the study period. Even a subject randomly assigned to this group may receive denosumab at its approved dosage as per its label if imaging data indicate the progression of bone erosion after entry into the study and if the investigator considers that the subject needs to receive denosumab.

### Use of co-interventions for the treatment of RA

All subjects of the study must continue to receive at least one csDMARD throughout the study period. During the study period, it will be permitted to switch from one to another csDMARD, to additionally prescribe any new csDMARDs, to discontinue a part of the csDMARD therapy, and to modify the dosage of the csDMARD therapy within the range of their approved dosages in Japan. During study period, the following treatments are prohibited; administration of bDMARDS or JAK inhibitors,, concomitant use of oral corticosteroids equivalent to more than 10 mg/day. Parenteral corticosteroid except intra-articular corticosteroid injections at joints other than those assessed in this study is prohibited. The concomitant use of a non-steroidal anti-inflammatory drug (NSAID) and an oral corticosteroid equivalent to not more than 10 mg/day of prednisolone will be permitted if this is considered necessary in light of the severity of symptoms in a specific subject. During the study period, the dosage of any NSAID can be modified within the range of its approved doses in Japan. Also, the dosage of any oral corticosteroid can be modified within the range of doses equivalent to not more than 10 mg/day of prednisolone.

### Adverse events

All adverse events (AEs) that occur between the administration of denosumab and the end of month 12 will be recorded. If necessary, the investigators will administer treatments. A serious AE (SAE) is defined as any adverse reaction resulting in any of the following outcomes: a life-threatening condition or death, a condition that requires inpatient hospitalization or prolongation of an existing hospitalization, threatening to cause disability or disability, a congenital anomaly, or a birth defect. Any SAEs will be documented in the medical records and be reported to the IRB by the responsible investigator in accordance with Japanese regulations.

### Outcome measurements

**Fig. 2:**
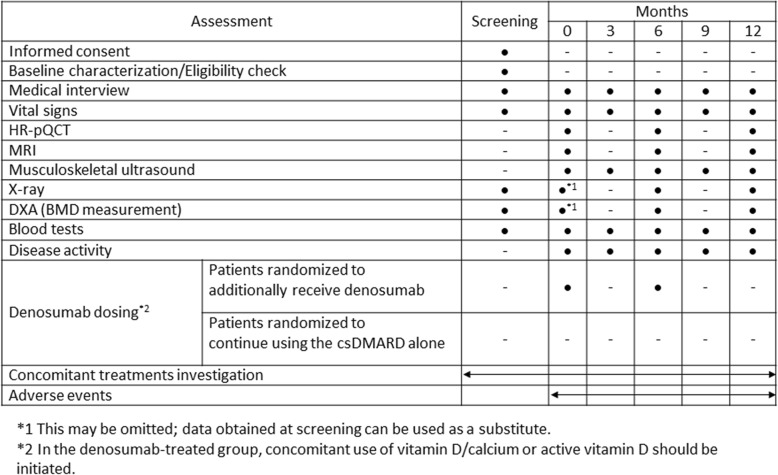
Treatment schedule and outcome measures. *Abbreviations*: *BMD* bone mineral density, *csDMARD* conventional synthetic disease-modifying anti-rheumatic drug, *DXA* dual-energy x-ray absorptiometry, *HR-pQCT* high-resolution peripheral quantitative computed tomography, *MRI* magnetic resonance imaging.

Study visits will take place at baseline and after 3, 6, 9, and 12 months. The assessments are represented in Fig. 2. HR-pQCT (XtremeCTII; Scanco Medical AG, Brüttisellen, Switzerland) of the second and third metacarpal bones and the wrist joint—of the affected hand, the more severely affected hand (if both hands are affected), or the hand of the subject’s dominant arm (if both hands are unaffected or affected to equal extents)—and of the left distal tibia will be performed at months 0, 6, and 12. In addition to estimating bone erosion, we will measure bone micro-architecture such as the trabecular volumetric bone mineral density (Tb. vBMD), trabecular bone volume fraction (BV/TV), trabecular number (Tb. N), trabecular thickness (Tb. Th), trabecular separation (Tb. Sp), structure model index (SMI), cortical volumetric bone mineral density (Ct. vBMD), cortical thickness (Ct. Th), and cortical porosity (Ct. Po.) by using HR-pQCT. Physical examination, determination of DAS28, and safety measures, including lab examinations, will be performed at every visit. Musculoskeletal ultrasound (AplioXG; Toshiba Medical Systems Corporation, Tochigi, Japan) of both hands will be taken every three months. Radiographs of both hands and feet and MRIs of the wrist and finger joints of the same hand assessed by HR-pQCT will be taken at baseline and after 6 and 12 months. Dual-energy x-ray absorptiometry (DXA) (Prodigy; GE Healthcare, Little Chalfont, UK) of the lumbar spine and hip at baseline and after 6 and 12 months is planned. Radiographs of the thoracic and lumbar spine will be taken for the detection of pre-existing vertebral fractures and new fractures at the same time as a bone mineral density (BMD) measurement by DXA.

The primary endpoint is the change from baseline in the depth of bone erosion as measured by HR-pQCT in the second and third metacarpal bones at 6 months after the start of treatment. The secondary endpoints consist of other findings measured by HR-pQCT. These include changes from baseline in the width and volume of bone erosion as measured by HR-pQCT in the second and third metacarpal bones and the wrist as well as changes from baseline in the depth of bone erosion in the second and third metacarpal bones at 12 months and changes from baseline in the depth of bone erosion in the wrist. The safety endpoint is incidence of the AEs.

### Exploratory endpoints

For the further evaluation of the efficacy of denosumab in RA, we will assess disease activity as defined by DAS28 and assess joint destruction by using other modalities such as MRI and HR-pQCT. The exploratory endpoints of this study are summarized in Table 1.

**Table 1:** Exploratory endpoints of this study

1) HR-pOCT measurements	
(1) Measurements in the second and third metacarpal bones and in the wrist joint • Changes from baseline in periarticular BMD • Changes from baseline in periarticular bone micro-architecture • Changes from baseline in the width of joint space • Changes from baseline in BMD at the rim of erosive lesions (2) Measurements in the tibial bone • Changes from baseline in BMD • Changes from baseline in bone micro-architecture	
2) Magnetic resonance imaging measurements	
• Changes from baseline in the extent of osteitis • Changes from baseline in the extent of bone erosion • Changes from baseline in synovitis score	
3) Musculoskeletal ultrasound measurements	
• Changes from baseline in synovitis score • Changes from baseline in the extent of bone erosion	
4) X-ray measurements	
• Changes from baseline in bone erosion score • Changes from baseline in joint space narrowing score • Changes from baseline in the modified total Sharp score	
5) DXA measurements	
• Changes from baseline in lumbar spine BMD • Changes from baseline in femoral BMD	
6) Changes from baseline in bone/cartilage turnover markers	
7) Changes from baseline in other biomarkers	
8) Changes from baseline in DAS28	

*Abbreviations*: *BMD* bone mineral density, *DAS28* disease activity score in 28 joints, *DXA* dual-energy x-ray absorptiometry, *HR-pQCT* high-resolution peripheral quantitative computed tomography.

### Data collection and management

All data are recorded in a case report form (CRF) by appropriate and authorized persons (investigator or clinical research coordinator). Only the patient number is recorded in the CRF. The investigator maintains a personal identification list (patient numbers with the corresponding patient names) to enable records to be identified. All study findings and documents will be regarded as confidential. During the study, authorized sponsor–investigators will make regular site visits to review protocol compliance, conduct source data verification, assess laboratory procedure, and ensure that the study is being conducted in accordance with protocol requirements.

### Sample size consideration and statistical analyses

In this study, changes in bone erosion metrics will be assessed in units of not subjects but lesions and on the assumption that a single subject may have multiple lesions. It is estimated, on the basis of the results of a previous study [9], that the between-group difference in changes at 6 months in the depth of bone erosion is 1.0 mm and that the standard deviation of the changes at 6 months is 1.1 mm. Given the conservative assumption that one bone erosion lesion is evaluable per subject, the smallest number of subjects required for detecting the effect size of treatment at a power of 80% and a two-tailed significance level of 0.05 is calculated to be 42 (21 per group). Moreover, the statistical power is stronger when assuming that a single subject has more than one bone erosion lesion and that two lesions are evaluable per subject (Table 2). Given the potential dropout of a few subjects and an increase in the standard deviation, an enrollment of 44 subjects is planned, as this sample size will provide adequate statistical power.

**Table 2:** Statistical powers provided by a sample size of *n* = 42 (21 per group) if two lesions are evaluable per subject

Effect size (between-group difference)	1.0 mm					
Standard deviation	1.1 mm			1.3 mm		
Intra-subject correlation	0.1	0.3	0.5	0.1	0.3	0.5
Statistical power	97.1%	94.5%	91.5%	90.8%	85.8%	80.1%

### Statistical analysis method

Efficacy analysis will be carried out on the full analysis set (FAS) and per protocol set (PPS). The FAS will consist of all enrolled patients who have HR-pQCT measurements available both at baseline and at 6 months. The PPS is defined as a subset of the FAS which excludes patients with major protocol violations and thus represents greater compliance with the protocol.

As for the baseline characteristic data, data on categorical variables will be summarized by group in frequency tables. Data on continuous variables will be summarized by group in terms of summary statistics.

The FAS will be the primary population analyzed for the primary endpoint: changes from baseline in the depth of bone erosion as measured by HR-pQCT at 6 months after the start of treatment. The mean change and standard deviation will be calculated by treatment group. In addition, a linear mixed model involving treatment, randomization factors, and disease activity as the fixed effects, patient as the random effect, and the baseline depth of bone erosion as a covariate will be used to calculate the estimated between-group difference in adjusted means (csDMARDs + denosumab group minus csDMARDs alone group) with its 95% confidence interval (CI) and the *P* value. Similar analyses will be performed separately on the PPS. Data on changes from baseline in the width and volume of bone erosion (secondary endpoints) will be analyzed in a similar manner.

Safety endpoints will be analyzed in the safety analysis set, which will consist of all randomly assigned patients. AE data will be summarized by treatment group in frequency tables. Data on categorical laboratory parameters obtained at baseline and at each subsequent measurement will be displayed by treatment group in cross-frequency tables. For continuous laboratory parameters, the values observed and changes from baseline at each specified time will be summarized by treatment group as summary statistics. Unless otherwise specified, all tests of significance will be carried out at a two-tailed significance level of 0.05, and inferences will be made by using two-tailed 95% CI.

## Discussion

The aim of this trial is to examine the inhibitory effect on bone erosion progression of denosumab treatment in subjects with RA with low or moderate disease activity. In order to adequately evaluate the effect of denosumab, continued conventional treatment without denosumab is used as an open-label randomized control. We set the current use of bDMARDs or JAK inhibitors as an exclusion criterion for the following reasons. The inhibitory effect of these treatments on the progression of joint destruction is obvious from many clinical trials and even from post-marketing surveillances and retrospective large cohort studies [10, 11]. Therefore, the treatment with bDMARDs or JAK inhibitors may strongly affect the efficacy, and we cannot evaluate the effect of denosumab. Moreover, given the clinical use of denosumab in practice, the concomitant use of bDMARDs/JAK inhibitors with denosumab may be relatively rare as compared with the concomitant use of csDMARDs because bDMARDs/JAK inhibitors are solely effective in the treatment of RA.

In this trial, we will use HR-pQCT to evaluate bone micro-architecture and bone erosion. Several studies have investigated the severity of bone erosion by using HR-pQCT in patients with RA [6, 12, 13]. One of these studies, an open-label, randomized, parallel-group study conducted by Yue *et al*., compared denosumab with alendronate, a bisphosphonate, in 40 women with RA and low bone mass [13]. In the post-hoc analysis of the study, Yue *et al*. analyzed the progression of bone erosion as measured by HR-pQCT. After 6 months of treatment, patients who received denosumab showed a significantly reduced size of bone erosion from baseline whereas patients who received alendronate had a significantly increased size of bone erosion from baseline. Thus, this previous study has shown the efficacy of denosumab in suppressing the progression of bone erosion, although this line of evidence is at a low level of certainty because of the post-hoc nature of the analysis. Another limitation of the study was the involvement of patients with low bone mass who had previously not received treatment for osteoporosis; that is, the patient population was very selective.

In addition to bone erosion, bone micro-architecture can be assessed by HR-pQCT. Periarticular osteoporosis is one hallmark of RA which is included in the Steinbrocker stage score [14]. Several studies revealed that periarticular osteoporosis assessed by digital x-ray radiogrammetry was related to bone erosion [15, 16]. However, until now, the periarticular bone structure, including BMD, has not been fully elucidated. Because this trial uses HR-pQCT to assess bone micro-architecture, it has the potential to elucidate periarticular osteoporosis in RA during the disease course and to elucidate the pathogenesis of RA from the point of view of bone structure.

We will evaluate synovitis by musculoskeletal ultrasound and detect osteitis by using MRI. In the presence of RA-related joint inflammation, various cytokines are activated in inflamed joints, and the activated cytokines induce the expression of RANKL at high levels, promoting bone destruction. Because the suppression of bone destruction progression by csDMARDs is mediated by their anti-inflammatory effect, which suppresses RANKL expression, the suppression of bone erosion can occur in parallel with the suppression of synovitis. In contrast, denosumab could suppress the progression of bone destruction by a mechanism independent of its ability to control inflammation. Therefore, the suppression of bone erosion by denosumab is expected to occur without being associated with the suppression of synovitis, although this remains to be confirmed in clinical studies. Also, osteitis, a term which means inflammation within the bone marrow, has been suggested to have the potential to cause bone erosion. More osteoclasts are present and a higher level of RANKL is expressed in bones with osteitis than in those without osteitis [17]. By taking an MRI, musculoskeletal ultrasound, and HR-pQCT at almost the same time point, we will be able to examine the effect of denosumab on osteitis, synovitis, and bone erosion and the relations between them.

In this trial, we will evaluate the efficacy and safety of denosumab in RA patients receiving csDMARDs. Previous clinical trials have shown the benefits of denosumab in inhibiting the progression of bone erosion. In practice, however, it is important to show the benefits of denosumab compared with conventional therapy as well as with a placebo. Evidence for the greater suppression of bone erosion progression by denosumab compared with conventional therapy will provide a basis for selecting denosumab for the management of RA in practice. By using HR-pQCT, the study will also be able to answer other clinical questions, possibly contributing to the progress of medicine and science.

### Trial status

The first version was published on January 16, 2018, and was last updated on September 11, 2018. Recruitment started in March 2018 and is expected to finish in 2021.

## Additional file

Additional file 1: SPIRIT (Standard Protocol Items: Recommendations for Interventional Trials) 2013 Checklist: Recommended items to address in a clinical trial protocol and related documents*. (DOC 120 kb)

## Data Availability

The datasets used or analyzed (or both) during the current study are available from the corresponding author on reasonable request.

## Abbreviations

ACR: American College of Rheumatology
AE: Adverse event
bDMARD: Biologic disease-modifying anti-rheumatic drug
BMD: Bone mineral density
CI: Confidence interval
CRF: Case report form
csDMARD: Conventional synthetic disease-modifying anti-rheumatic drug
DAS28: Disease activity score in 28 joints
DXA: Dual-energy x-ray absorptiometry
FAS: Full analysis set
HR-pQCT: High-resolution peripheral quantitative computed tomography
IRB: Institutional review board
JAK: Janus kinase
MRI: Magnetic resonance imaging
NSAID: Non-steroidal anti-inflammatory drug
PPS: Per protocol set
RA: Rheumatoid arthritis
RANK: Receptor activator of nuclear factor kappa B
RANKL: Receptor activator of nuclear factor kappa B ligand
SAE: Serious adverse event
